# Supplementary material for: The Effect of Schisandra chinensis Baillon on Cross-Talk between Oxidative Stress, Endoplasmic Reticulum Stress, and Mitochondrial Signaling Pathway in Testes of Varicocele-Induced SD Rat
Source: Int J Mol Sci. 2019 Nov 17;20(22):5785. doi: 10.3390/ijms20225785 (PMC6888522; doi:10.3390/ijms20225785)
Supplement: Supplementary file 1 [file ijms-20-05785-s001.zip › Supplementary Table 2.docx]

**Supplementary Table 2**

Linear regression data, LOD and LOQ of investigated schisandrol A in SC.

| **Compound** | Linear regression data | | | LOD | LOQ |
| --- | --- | --- | --- | --- | --- |
|  | Regressive equation | Test range (mg/L) | r^2^ |  |  |
| **Schisandrol A** | y = 20,222 x + 53665 | 5 - 200 | 1.0000 | 0.3319 | 0.1006 |

LOC: limit of detection; LOQ: limit of quantification.
