# Supplementary material for: The Effect of Schisandra chinensis Baillon on Cross-Talk between Oxidative Stress, Endoplasmic Reticulum Stress, and Mitochondrial Signaling Pathway in Testes of Varicocele-Induced SD Rat
Source: Int J Mol Sci. 2019 Nov 17;20(22):5785. doi: 10.3390/ijms20225785 (PMC6888522; doi:10.3390/ijms20225785)
Supplement: Supplementary file 1 [file ijms-20-05785-s001.zip › Supplementary Table 3.docx]

**Supplementary Table 3**

Intra- and inter-day variability for the assay of investigated schisandrol A in SC.

| **Compound** | Concentration  (mg/L) | Accuracy  (bias, %) | | Precision  (^***^c.v., %) | |
| --- | --- | --- | --- | --- | --- |
|  |  | ^*^Intra-day | ^**^Inter-day | ^*^Intra-day | ^**^Inter-day |
| **Schisandrol A** | 5 | 103.81 | 101.24 | 1.00 | 2.70 |
|  | 100 | 100.48 | 101.31 | 0.13 | 1.94 |
|  | 200 | 99.90 | 100.23 | 0.49 | 1.14 |

(^*^Intra-day: three times per day, ^**^Inter-day: one times analysis of standards per day for three days, ^***^c.v.: Co-effecinet of variation.)
