# Supplementary material for: The Effect of Schisandra chinensis Baillon on Cross-Talk between Oxidative Stress, Endoplasmic Reticulum Stress, and Mitochondrial Signaling Pathway in Testes of Varicocele-Induced SD Rat
Source: Int J Mol Sci. 2019 Nov 17;20(22):5785. doi: 10.3390/ijms20225785 (PMC6888522; doi:10.3390/ijms20225785)
Supplement: Supplementary file 1 [file ijms-20-05785-s001.zip › Supplementary Table 4.docx]

**Supplementary Table 4**

Content of schisandrol A in SC.

| **Sample** | **Schisandrol A** |
| --- | --- |
| *Schisandra chinensis* Baillon 100% EtOH ex. | 2.915% |
