# Supplementary material for: The Effect of Schisandra chinensis Baillon on Cross-Talk between Oxidative Stress, Endoplasmic Reticulum Stress, and Mitochondrial Signaling Pathway in Testes of Varicocele-Induced SD Rat
Source: Int J Mol Sci. 2019 Nov 17;20(22):5785. doi: 10.3390/ijms20225785 (PMC6888522; doi:10.3390/ijms20225785)
Supplement: Supplementary file 1 [file ijms-20-05785-s001.zip › Supplementary Table 5.docx]

**Supplementary Table 5**

The effect of five compounds in SC on sperm motility in human semen sample.

| Group | Patient 1` | | | | Increase  in sperm motility  (%) | Patient 2` | | | | Increase  in sperm motility  (%) | Patient 3` | | | | Increase  in sperm motility  (%) |
| --- | --- | --- | --- | --- | --- | --- | --- | --- | --- | --- | --- | --- | --- | --- | --- |
|  | Zero | | 3h | |  | Zero | | 3h | |  | Zero | | 3h | |  |
| Compound | Count  (10^6^/mL) | Motility  (%) | Count  (10^6^/mL) | Motility  (%) |  | Count  (10^6^/mL) | Motility  (%) | Count  (10^6^/mL) | Motility  (%) |  | Count  (10^6^/mL) | Motility  (%) | Count  (10^6^/mL) | Motility  (%) |  |
| Control | 60 | 42 | 52 | 46 | 9.5 | 38 | 61 | 33 | 53 | -13.1 | 39 | 50 | 32 | 43.2 | -13.6 |
| **Schisandrol A (1)**  **10 μM** | 53 | 43.5 | 53 | 58.2 | **33.8** | 41 | 60 | 37 | 62.4 | **4** | 34 | 45.8 | 35 | 54.3 | **18.6** |
| Schisandrol B (2)  10 μM | 55 | 40 | 50 | 47.4 | 18.5 | 48 | 61.2 | 35 | 50 | -18.3 | 35 | 46.2 | 32 | 40 | -13.4 |
| Schisandrin A (3) 10 μM | 52 | 45.2 | 49 | 48.3 | 6.9 | 37 | 56 | 33 | 56.3 | 0.54 | 35 | 53.3 | 31 | 34.6 | -35.1 |
| Gomisin N (4)  10 μM | 60 | 42 | 49 | 46.5 | 10.7 | 41 | 65 | 28 | 54.4 | -16.31 | 39 | 50.5 | 24 | 38.8 | -23.2 |
| Schisandrin C (5) 10 μM | 61 | 45 | 50 | 52.7 | 17.1 | 38 | 62.6 | 32 | 55.5 | -11.3 | 36 | 50 | 21 | 40 | -20 |
