# Supplementary material for: The Effect of Schisandra chinensis Baillon on Cross-Talk between Oxidative Stress, Endoplasmic Reticulum Stress, and Mitochondrial Signaling Pathway in Testes of Varicocele-Induced SD Rat
Source: Int J Mol Sci. 2019 Nov 17;20(22):5785. doi: 10.3390/ijms20225785 (PMC6888522; doi:10.3390/ijms20225785)
Supplement: Supplementary file 1 [file ijms-20-05785-s001.zip › Supplementary Table 1.docx]

**Supplementary Table 1**

The effects of SC extract on hematology and clinical chemistry parameters in VC-induced male SD rats.

| Parameters | CTR | SC 200 | VC | VC + SC 200 |
| --- | --- | --- | --- | --- |
| RBC (×10^4^/ μL) | 7.92 ± 0.13 | 7.64 ± 0.08 | 7.83 ± 0.07 | 7.89 ± 0.07 |
| WBC (×10^3^/ μL) | 8.38 ± 0.31 | 7.35 ± 0.35 | 8.86 ± 0.64 | 8.00 ± 0.34 |
| HB (g/dL) | 14.10 ± 0.12 | 13.91 ± 0.14 | 13.96 ± 0.12 | 14.19 ± 0.19 |
| HCT (%) | 43.41 ± 0.36 | 42.87 ± 0.50 | 43.13 ± 0.47 | 44.25 ± 0.20 |
| Platelet (X10^3^/µl) | 557.40 ± 47.67 | 727.80 ± 17.66 | 720.60 ± 21.62 | 665.10 ± 26.96 |
| AST (IU/L) | 120.70 ± 13.37 | 110.90 ± 9.80 | 129.00 ± 8.05 | 100.10 ± 5.39 |
| ALT (IU/L) | 52.10 ± 4.79 | 46.20 ± 3.75 | 51.40 ± 6.35 | 48.40 ± 2.86 |

Data were presented in mean ± S.E.M, n = 10. Statistical analyses were performed using one-way ANOVA followed by Tukey’s post hoc test. CTR, control; SC 200, SC 200 mg/kg p.o; VC, varicocele; VC + SC 200, SC 200 mg/kg; SC, Schisandra chinensis; LH, luteinizing hormone; FSH, follicle stimulating hormone; WBC, white blood cell; RBC, red blood cell; Hb, hemoglobin; Hct, hematocrit; AST, aspartate aminotransferase; ALT, alanine aminotransferase; p.o., per oral; ANOVA, analysis of variance; SEM, standard error of the mean
